# New Tetramic Acids Comprising of Decalin and Pyridones From *Chaetomium olivaceum* SD-80A With Antimicrobial Activity

**DOI:** 10.3389/fmicb.2019.02958

**Published:** 2020-01-15

**Authors:** Xinzhu Wang, Liya Zhao, Chao Liu, Jun Qi, Peipei Zhao, Zhaoming Liu, Chunlei Li, Yingying Hu, Xin Yin, Xin Liu, Zhixin Liao, Lixin Zhang, Xuekui Xia

**Affiliations:** ^1^Department of Pharmaceutical Engineering, School of Chemistry and Chemical Engineering, Southeast University, Nanjing, China; ^2^Shandong Provincial Key Laboratory for Biosensor, Biology Institute, Qilu University of Technology (Shandong Academy of Sciences), Jinan, China; ^3^Institute of Agro-Food Science and Technology, Shandong Academy of Agricultural Sciences, Jinan, China; ^4^Guangdong Institute of Microbiology, Guangdong Academy of Sciences, Guangzhou, China; ^5^State Key Laboratory of Bioreactor Engineering, East China University of Science and Technology, Shanghai, China

**Keywords:** *Chaetomium olivaceum*, tetramic acids, isolation, anti-MRSA, biosynthesis pathway

## Abstract

Cycloaddition reactions such as intramolecular Diels–Alder (IMDA) are extremely important in constructing multicyclic scaffolds with diverse bioactivities. Using MycB as a biomarker, three new polyketides – Chaetolivacines A (**1**), B (**3**), and C (**4**) – with one known compound Myceliothermophin E (**2**) comprising of decalin and 4-hydroxy-2-pyridones were obtained from the culture of *Chaetomium olivaceum* SD-80A under the guidance of gene mining. The structures of these compounds were established using detailed 1D, 2D NMR, and high-resolution electron spray ionization mass spectroscopy (HRESIMS) analysis. The relative and absolute configurations of the compounds **1**, **3**, and **4** were elucidated by NOESY and ECD. The biosynthesis pathways of these compounds were proposed, which involves in three key genes *ChaA* [polyketide synthase-non-ribosomal peptide synthetases (PKS-NRPS)], *ChaB*, and *ChaC*. Compounds **1**–**4** were tested for their antimicrobial activities, and compounds **2** and **3** showed moderate bioactivity against *Staphylococcus aureus* (SA) and methicillin-resistant *S. aureus* (MRSA) with MIC values of 15.8 and 27.1 μM. The results showed that configuration of C-21 in **3** and **4** is important for anti-SA and anti-MRSA activities. This study reveals the significant potential of the genus *Chaetomium* in producing new PKS-NRPS, therefore increasing the speed in the mining for new sources of antimicrobial agents.

## Introduction

The increasing resistance to drugs of bacteria like methicillin-resistant *Staphylococcus aureus* (MRSA) has become a major threat to public health (Coast et al., 1996; Stanton, 2013). The first strain of MRSA appeared in Cairo in 1961. Since then, the specific strain has spread to become a worldwide problem (Chapman et al., 2005). MRSA has increased in prevalence during the past decade due to the steady growth of elderly and immunocompromised patients and the emergence of multidrug-resistant (MDR) bacterial strains. Because MRSA is one of the most common and problematic bacteria associated with increasing antimicrobial resistance, continuous efforts to discover compounds, develop alternative therapies, and create faster diagnostics methods are required (Kurosu et al., 2013). MRSA continues to be associated with significant morbidity and mortality rates. Vancomycin was the “gold standard” for treatment of serious MRSA infections; however, the emergence of less-susceptible strains, poor clinical outcomes, and increased nephrotoxicity associated with high-dose therapy have challenged vancomycin’s role as a first-line therapy. Linezolid is recommended for PO or IV treatments of skin and skin structure infections (SSSIs) and pneumonia caused by MRSA. Daptomycin (IV) should be considered for patients with MRSA bacteremia and right-sided endocarditis as well as for cases of complicated SSSIs; however, daptomycin should not be used to treat MRSA-related pneumonia. Tigecycline and telavancin are alternative (IV) treatments for SSSIs caused by MRSA; however, safety concerns have limited use of these agents (Rodvold and McConeghy, 2013). Recently, more and more attention has been devoted to fungi, which is an important resource to produce lead compounds for drug-resistant bacteria (Ghisalberti, 2003). *Chaetomium* sp. is a large fungal group (Royles, 1995) known for its secondary metabolites with physiological activity and is considered a potential biological control group (Hazuda et al., 1999; Kirk et al., 2001). A variety of secondary metabolites can be produced by the *Chaetomium* sp., such as decalinepolyketides containing a tetramic acid (2,4-pyrrolidine-2,4-dione) ring; it is one of the most important classes of metabolites (Yang et al., 2006, Yang S.W. et al., 2007). The tetramic acid (2,4-pyrrolidine-dione) ring system has been reported since the early 20th century (Osterhage et al., 2000) when the first simple derivative was prepared. Compounds containing this specific structural unit exhibit a diverse range of biological activities (Soytong et al., 2001). For instance, codinaeopsin (Kontnik and Clardy, 2008; Ramanathan et al., 2013) is active against *Plasmodium falciparum*, the causative agent for the most lethal form of malaria. Equisetin (Vesonder et al., 1979; Wheeler et al., 1999) suppresses germination or inhibits the growth of various monocotyledonous and dicotyledonous seeds. Additionally, it inhibits the growth of young seedlings and causes necrotic lesions on the roots, cotyledons, and coleoptiles of tested plant seedlings. Trichosetin (Marfori et al., 2002a, 2003b) is produced by the dual culture of *Trichoderma harzianum* and *Catharanthus roseus* callus and shows remarkable antimicrobial activity against the Gram-positive bacteria MRSA and *Bacillus subtilis*. UCS1025A (Nakai et al., 2000) exhibited antimicrobial and antiproliferative activities.

Many important compounds are produced by polyketide synthase-non-ribosomal peptide synthetase (PKS-NRPS) hybrid clusters, such as fusarin C (Song et al., 2004), equisetin (Kato et al., 2015), and decalinepolyketides (Han et al., 2017). This type of compounds exhibit a wide variety of biological activities (Boettger and Hertweck, 2012; Chooi and Tang, 2012). In recent years, with the development of whole genome sequencing technology, an increasing number of genomes have been released. The separation of natural products, which were biosynthesized by the regulation of particular gene clusters, has become more efficient under the guidance of genome mining. According to recent studies, collecting and mining “Diels–Alderases” (DAases) from fungi may lead to the discovery of new decalin-containing natural products (Li et al., 2016). Based on the above literature, the *trans*-decalin tetramic acids structure is widely present in bioactive natural products isolated from fungi. In this study, under the guidance of biosynthesis gene cluster analysis of *C. olivaceum* SD-80A, four derived decalin-containing natural products were obtained. Apart from myceliothermophin E (**2**) (Yang Y.L. et al., 2007; Shionozaki et al., 2012), the compounds **1**, **3**, and **4** are new. Their activity against drug-resistant bacteria was tested for compounds **1**–**4**. Compounds **2** and **3** showed moderate bioactivity against MRSA (MIC = 15.8 and 21.7 μM, respectively). In conclusion, we report the detailed isolation, structural elucidation, biological activities, and possible biosynthesis pathways of these compounds. This is also the first time that these compounds have been found to exhibit anti-MRSA activity.

## Materials and Methods

### Genome Mining of Biosynthetic Gene Clusters

The genome *C. olivaceum* SD-80A was firstly deciphered using *de novo* sequencing technology. As reported, DAases have many important biological functions (Zheng et al., 2016). The diversity of DAases have been found to simulate the discovery of certain structural features compounds (Li et al., 2016). In this work, MycB (NCBI Accession No. AEO57198), a kind of DAases used as a signature biosynthetic marker from *Myceliophthora thermophila* was used as probe for mining the homologous protein based on the *C. olivaceum* SD-80A genome. Gene cluster analysis was implemented using anti-SMASH 3.0 (Weber et al., 2015), and gene function was predicted by NCBI combined with reported references (Doroghazi et al., 2014). Amino acid sequence alignment was performed by BLAST^1^.

### Producing Fungus and Fermentation

The fungus *C. olivaceum* SD-80A was isolated from a fece of *Boselaphus tragocamelus* collected in New Delhi, India. The strain was grown on a PDA medium for 7 days in the dark at 28°C, then transferred to five 500 mL Erlenmeyer flasks with a PDB medium for 5 days at 28°C, with 120 rpm as the seed medium. After the seed medium grew well, the culture was evenly transplanted to 30 1 L Erlenmeyer flasks on an ultra-clean bench, each containing 50 mL of water and 50 g of rice. Fermentation was conducted under stationary conditions for 30 days at 28°C. After the fermentation, we procured the fungus of *C. olivaceum* SD-80A.

### Solvent Extraction and Purification of Secondary Metabolites

The fungus *C. olivaceum* SD-80A was extracted with ethyl acetate (5 × 2 L, 24 h each time) at 25°C to create a dark brown residue (12.0 g) after removal of the solvent *in vacuo*. The ethyl acetate extract (12.0 g) was subjected to a silica gel column using petroleum ether-EtOAc (100:0–0:100, v/v) as an eluent to create seven fractions (Fr. A–G). Fr. A (0.6 g) was separated with semi-preparative high-performance liquid chromatography (HPLC) using CH_3_OH-H_2_O (60:40–100:0, v/v) as the mobile phase to create Fr. A.1 (306.7 mg). Fr. A.1 was further followed by semi-preparative HPLC by using CH_3_OH-H_2_O (80:20–100:0, v/v) as the mobile phase to create **1** (22.5 mg) and **2** (48.6 mg). Fr. C (2.8 g) was subjected to a silica gel column using petroleum ether-EtOAc (15:85–0:100, v/v) to generate Fr. C.1-C.6. Fr. C.3 was separated by semi-preparative HPLC eluted by CH_3_OH-H_2_O (80:20–100:0, v/v) to yield compounds **3** (8.1 mg, *Rt* = 15.0 min) and **4** (9.7 mg, *Rt* = 17.6 min) (Supplementary Figure S25).

### Antimicrobial Activity Assay of Fractions

All antimicrobial assays mentioned in the article were performed using Gram-positive bacteria pathogens *S. aureus* (SA, ATCC 6538) and MRSA (clinical strain from Chaoyang Hospital, Beijing, China) to which vancomycin was used as a positive control, and Gram-negative bacteria *Pseudomonas aeruginosa* strain 14 (PA14, ATCC 15692) to which ciprofloxacin was used as a positive control. For each organism, a loopful of glycerol stock was streaked on an LB-agar plate, which was incubated overnight at 37°C. The *Candida albicans* strains (type G5, 17#, ATCC 10231; type SC5314, ATCC MYA-2876) were grown on Sabouraud dextrose agar and incubated overnight at 28°C; Amphotericin B was used as a positive control.

The fresh (about 3 days old) single colony bacteria were removed from the YPD medium plate with an inoculating ring and mixed in 1 mL RPMI1640 medium. The concentration of bacterial solution was then properly adjusted. In a 96-well plate, an 80 μL bacterial solution of the above concentration was added to each well; the first column was added with antibiotics as the positive control, and the last column was added with DMSO as the negative control. The compound was then added to be tested in the middle. During the operation, the concentration of a compound in the intermediate part was successively half diluted by the pipette. In this study, all isolated compounds (**1**–**4**) were screened for their antibacterial activity and antibiotic resistance bacteria activity toward a panel of bacteria using the MIC method.

### Experimental Apparatus and Materials

The 1D and 2D NMR spectra were recorded in CDCl_3_ on a BRUKER ASCEND 400 AVANCE III HD spectrometer with tetramethylsilane as the internal standard. All NMR assignments were based on the ^1^H-^1^H COSY, HSQC, HMBC, and NOESY spectroscopic data. High-resolution electron spray ionization mass spectroscopy (HRESIMS) data were acquired on an Agilent 6520 Q-TOF mass spectrometer (Agilent Technologies, United States). ECD spectra were recorded on a JASCO J-815 spectropolarimeter using a Nicolet Magna-IR 750 spectrophotometer. Sephadex LH-20 (Pharmacia, Sweden), MCI gel (CHP20P, 75-150 μm, Mitsubishi Chemical Industries Ltd.) and YMC ODS-AQ (S-50 μm, 12 nm, YMC Co., Ltd., Japan). Thin-layer chromatography was performed on a precoated GF254 plate (Qingdao Marine Chemical Co., Ltd., China). Spots were detected under UV light followed by heating after spraying with a 10% vanillin/H_2_SO_4_ solution. Semi-preparative HPLC was carried out on an Agilent 1260 Infinity instrument equipped with a DAD detector and a Waters Sunfire Prep C-18 column (10 × 250 mm, 5 μm).

### Genome Analysis and Functional Assays

**FIGURE 1:**
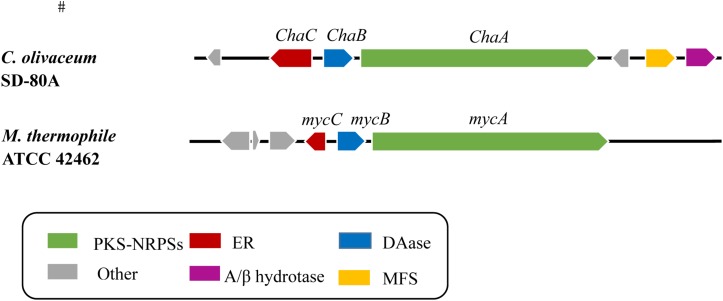
Comparison of *Cha* cluster and homologous gene cluster myc containing a PKS-NRPS hybrid, a DAse and a *trans*-ER. PKS-NRPS, polyketide synthase–non-ribosomal peptide synthetase; ER, enoylreductase; DAse, Diels–Alderases; MFS, major facilitator superfamily; α/β hydrotase, Alpha/beta hydrolase family.

To get a genome of *Chaetomium olivaceum* SD-80A, *de novo* whole genome sequencing approach was used and a 500 bp library was sequenced on Illumina HiSeq 2500 platform. In this work, the total size of predicted assembly is 34782639 bp. The resulting 3.47 G of data were assembled to 10658 scaffolds using the SOAP *de novo* (v2.04) assembler (Luo et al., 2012). The average G + C content is 55.57% (Table 1). The amino acid sequence of MycB (XP_003662443.1) was used as a probe to blast the genome of *C. olivaceum* SD-80A. Four homologous gene sequences that were separately located at scaffold 1389, scaffold 1605, scaffold 647, and scaffold 1433 were mined with different levels of identities of 68, 32, 26, and 28%, respectively. Herein, the scaffold 1389 has a higher identity and the *E*-value is 0. Therefore, it is a likely candidate. Furthermore, the identity between the homologous protein encoded by the gene at scaffold 1389 and MycB was up to 68%. The corresponding gene was designated as *ChaB*. Combined with the gene cluster analysis results of the *C. olivaceum* SD-80A genome, *ChaB* and a PKS-NRPS gene located at scaffold 1389 were adjacent (Figure 1). This PKS-NRPS gene showed an identity of 68% with the myceliothermophin gene (MycA, XP_003662442) from *M. thermophila* ATCC 42464 was named as *ChaA*. Through further sequence analysis, the MycC (XP_003662444) from *M. thermophila* ATCC 42464 has a 55% identity sequence homology to *ChaC*, another adjacent gene of *ChaB*. The presence of these three genes in the *C. olivaceum* SD-80A genome suggests that the strain may produce decalin-containing compounds during secondary metabolism.

**TABLE 1:** The features of the *Chaetomium olivaceum* SD-80A genome.

**Features**	***Chaetomium olivaceum* SD-80A**
Sequencing technology	*De novo* Illumina Hiseq
Total size	34,782,639
Average G + C content	55.57%
Scaffolds	10658

### Structural Analysis and Identification of Compounds

**FIGURE 2:**
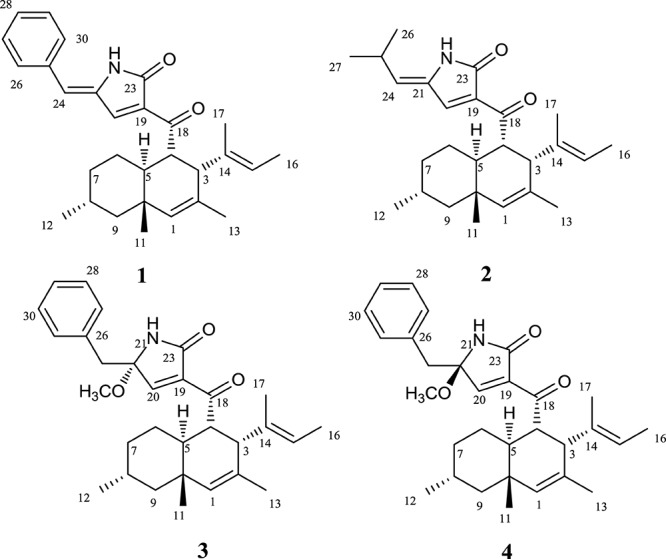
Sturctures of Compounds **1**–**4**.

To verify our inferences, secondary metabolites of *C. olivaceum* SD-80A were accumulated through fermentation. Four decalin-containing compounds were obtained as follows (Figure 2): Compound **1** was obtained as yellow amorphous powder, and its molecular formula was designated as C_29_H_35_NO_2_ according to the ion peaks at *m/z* 430.2266 [M + H]^+^ under its HRESIMS (Supplementary Figure S1), which indicates that it contains 12 degrees of unsaturation. The formula was supported by the ^1^H NMR and ^13^C NMR data. The ^1^H NMR spectrum (Table 2 and Supplementary Figure S2) exhibited one active hydrogen at δ_H_ 8.65 (22-NH), five CH_3_ at δ_H_ 0.90 (H-11), 0.86 (H-12), 1.48 (H-13), 1.43 (H-16), and 1.46 (H-17), four CH at δ_H_ 5.37 (H-1), 5.08 (H-15), 7.61 (H-20), and 6.34 (H-24), five benzene ring CH at δ_H_ 7.50 (H-26, H-30), 7.45 (H-27, H-29), and 7.35 (H-28). The ^13^C NMR data (Table 2 and Supplementary Figure S3) exhibited 29 signals consisting of five methyls, three methylenes, 13 methines, and eight quaternary carbon atoms, including two carbonyl atoms at δ_C_ 169.8 and 196.6. Furthermore, δ_C_ 134.8 (C-19), 143.9 (C-20), 134.2 (C-21), and 169.8 (C-23) with the N atom could be elucidated to a pyridine ring.

**TABLE 2:** ^1^H and ^13^C NMR data of **1** and **2** (δ_H_ in ppm, *J* in Hz).

	**1^a^**		**2^a^**	
**Position**	**δ_C_**	**δ_H_, mult. (*J* in Hz)**	**δ_C_**	**δ_H_, mult. (*J* in Hz)**
1	136.3	5.37, s	136.1	5.36, s
2	130.2		131.7	
3	51.1	3.23, d (7.2)	51.0	3.23 d (7.2)
4	49.7	3.89, dd (12, 7.2)	49.6	4.04, dd (12, 7.2)
5	40.0	1.86, td (12, 2)	39.8	1.84, td (12, 2)
6	24.3	0.98, m 1.69, m	24.2	0.99, m 1.68, m
7	35.8	0.98, m 1.70, m	35.8	0.98, m 1.70, m
8	27.5	1.69, m	28.3	1.69, m
9	48.6	0.95, m 1.47, m	48.5	0.95, m 1.47, m
10	35.2		35.3	
11	22.8	0.90, s	20.6	0.94, s
12	20.6	0.86, d (6.0)	22.8	0.86, d (6.0)
13	22.2	1.48, s	22.2	1.47, s
14	135.9		135.9	
15	123.4	5.08, d (6.8)	123.2	5.08, d (6.8)
16	13.3	1.43, s	13.3	1.44, s
17	14.0	1.46, s	14.2	1.45, s
18	196.6		197.1	
19	134.8		134.8	
20	143.9	7.61, d (2.0)	142.0	7.44 d (2.0)
21	134.2		134.0	
23	169.8		170.3	
24	119.5	6.34, s	130.3	5.48, d (10.0)
25	129.5		27.5	2.93, m
26,30	129.4	7.50, d (7.2)	22.5	1.11, d (6.4)
27,29	129.4	7.45, t (8.0)	22.4	1.14, d (6.4)
28	129.0	7.35, m		
NH		8.65, brs		10.65, brs

*^*a*^Compounds ***1*** and ***2*** were measured at ^1^H NMR (400 MHz), and ^13^C NMR (100 MHz) with CDCl_3_ as solvent.*

**FIGURE 3:**
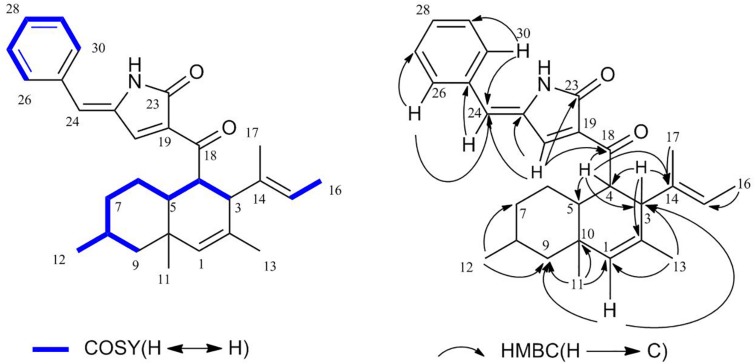
^1^H–^1^H COSY and Key HMBC correlations of **1**.

The ^1^H-^1^H COSY (Supplementary Figure S4) correlations of H-3, H-4, H-5, H-6, H-7, H-8, and H-9/12, correlations of H-15, H-16, correlations of H-26, H-27, H-28, H-29, and H-30 indicate three parts in blue color of **1** (Figure 3). The HMBC (HSQC and HMBC see Supplementary Figures S5, S6) correlations from H-1 to C-3, C-5, C-9, C-10, and C-13; from H-4 to C-3, C-5, and C-10; from 11-Me to C-1, C-5, and C-9, from 12-Me to C-7 and C-9; and from 13-Me to C-1 and C-3 revealed the presence of the decalin ring moiety. HMBC correlations from H-15 to C-16 and C-3 displayed a butene group linked to C-3. Furthermore, HMBC correlations of H-4, H-5, and H-20 to C-18 supported a tetramic acid moiety and the decalin ring joined with C-18. HMBC correlations of H-24 to C-29, C-21, C-26, and C-30 and correlations of H-26, H-20, and H-30 to C-24 indicate that the benzene connected to C-24.

**FIGURE 4:**
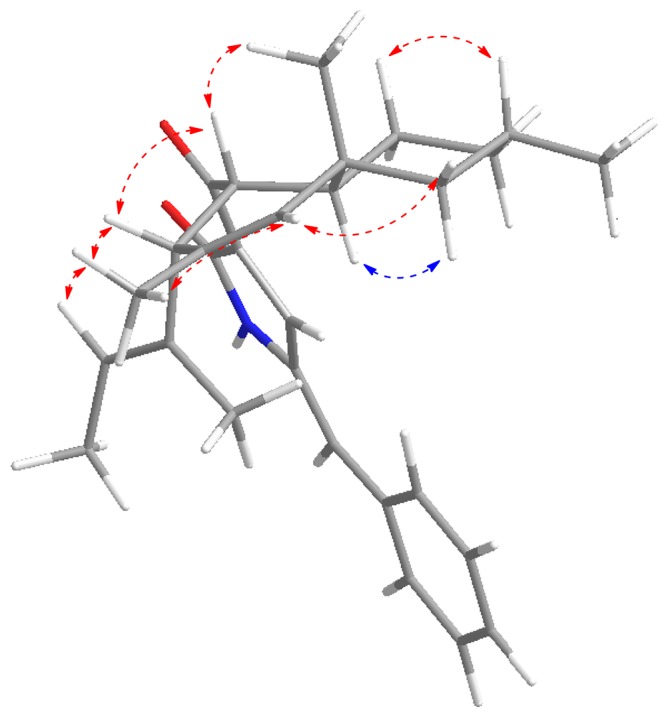
Key NOESY correlations of **1**.

A NOESY experiment (Supplementary Figure S7) combined with a coupling constant resulted in two 1,3-diaxial correlations. *J* = 12.0 Hz of H-4 and H-5 indicated the two protons are in an axial position. Additionally, H-4 was related to H-11 and H-5 was related to H-9α, which indicates that **1** has a *trans*-decalin ring (H-4/H-11 and H-5/H-9α). The other NOESY correlations from H-11 to H-6β, from H-6β to H-8, suggest that the orientation of CH_3_-12 is equatorial. In addition, it can also be elucidated from the NOESY correlation that H-3, H-4, H-8, and H-11 are β configurations, and H-5 is an α configuration. Thus, the relative configurations of **1** is determined as 3*S*^∗^4*R*^∗^5*R*^∗^8*R*^∗^10*S*^∗^ (Figure 4).

To establish the absolute configuration, ECD calculations were carried out to compare with the experiment plot. At first, the search for conformers was carried out by Spartan’14 with a Molecular Merck Force Field (MMFF). As a result, two conformers with an energy <10 kcal/mol were subjected to optimization to a minimum energy of b3lyp/6-31 + g(d, p), which consequently gave a Boltzmann distribution of 87.7 and 12.3%, respectively, based on their free energies (δ*G*). Secondly, the theoretical ECD plot in methanol was predicted at a b3lyp/6-311 + g(d, p) level and weighted with Boltzmann distribution. Solvent effects were taken into consideration using the self-consistent reaction field (SCRF) method with a polarizable continuum model (PCM). The calculated spectrum **1** showed an excellent fit with the experimental plot, suggesting that the absolute configuration should be 3*S*, 4*R*, 5*R*, 8*R*, and 10*S*. Finally, the overall characterization of compound **1** was elucidated and named as Chaetolivacine A. The experimental method is based on the experience of predecessors (Yang Y.L. et al., 2007).

The molecular formula of **2** was confirmed as C_26_H_37_NO_2_ through HRESIMS (Supplementary Figure S8), which has nine degrees of unsaturation. The ^1^H and ^13^C NMR data (Table 2 and Supplementary Figures S9, S10) were very similar to that of **1**, except that the monosubstituted benzene ring connected to C-24 in **1** was replaced by the isopropyl in **2**. Comparing the ^1^H NMR and ^13^C NMR data (Table 2) with that reported data, compound **2** was determined as myceliothermophin E (Yang Y.L. et al., 2007).

Compound **3** was isolated as yellow amorphous powders. The molecular formula of **3** was designated as C_30_H_39_NO_3_ according to the ion peaks at *m/z* 462.3036[M + H]^+^ in its HRESIMS (Supplementary Figure S11). The ^1^H NMR and ^13^C NMR data (Table 3 and Supplementary Figures S12, S13) of **3** are similar to that of **1**, except that the signal of one double bond at C-21/C-24 in **1** (δ_C__21_ 134.2, δ_C__24_ 119.5, δ_H__24_ 6.34, s) was reduced to C-21 (δ_C_ 89.8), C-24 (δ_C_ 44.2), H-24 (δ_H__24_ 3.12, d, *J* = 14.4 Hz, 3.02, d, *J* = 13.6 Hz), and a methoxy group OCH_3_-24 (δ_C_ 49.8) was connected to C-21. HMBC correlations from OCH_3_-24 to C-20, C-21 confirmed the elucidation. Then, the structure of **3** was elucidated and named as Chaetolivacine B.

**TABLE 3:** ^1^H and ^13^C NMR data of **3** and **4** (δ_H_ in ppm, *J* in Hz).

	**3^a^**		**4^a^**	
**No.**	**δ_C_**	**δ_H_, mult. (*J* in Hz)**	**δ_C_**	**δ_H_, mult. (*J* in Hz)**
1	136.4	5.35, brs	136.4	5.35, brs
2	136.3		136.3	
3	51.0	3.17, d (7.2)	51.0	3.06, d (7.6)
4	50.9	3.80, dd (12.4, 7.6)	51.0	3.83, dd (12.4, 7.6)
5	39.9	1.81 (dt, covered)	39.9	1.81 (dt, covered)
6	24.2	1.62, m 0.94, m	24.2	1.62, m 0.94, mF
7	35.8	1.69, m 0.95, m	35.7	1.69, m 0.95, m
8	27.4	1.60, m	27.4	1.60, m
9	48.5	1.48, m 0.97, m	48.5	1.48, m 0.97, m
10	35.1		35.2	
11	22.8	0.91, s	20.5	0.91, s
12	20.5	0.86, d (6.4)	22.8	0.86, d (6.4)
13	22.1	1.45, s	22.1	1.45, s
14	129.8		129.8	
15	123.0	5.02, dd (6.4)	123.1	5.07, dd (6.4)
16	13.1	1.41, s	13.3	1.41, s
17	13.7	1.39, s	13.7	1.39, s
18	196.0		196.4	
19	138.6		138.8	
20	154.3	7.40, d (1.6)	153.7	7.33, d (1.6)
21	89.8		89.7	
23	167.8		167.7	
24- O-CH_3_	49.8	3.13, s	49.9	3.13, s
25	44.2	3.12 (d, 14.4)	44.4	3.18 (d, 14)
		3.02 (d, 13.6)		2.95 (d, 13.6)
26	134.3		134.3	
27,31	130.6	7.23 (m)	130.5	7.23 (m)
28,30	128.4	7.28 (m)	128.5	7.28 (m)
29	127.4	7.30 (m)	127.4	7.30 (m)
NH		6.02, brs		5.86, brs

*^*a*^Compounds **3** and **4** were measured at ^1^H NMR (400 MHz) and ^13^C NMR (100 MHz) with CDCl_3_ as solvent. Chaetolivacine A (**1**), yellow amorphous powder, ${\left[\alpha \right]}_{D}^{20}$ = + 18.6 (c 0.1, MeOH); HRESIMS *m/z* 430.2266 [M + H]^+^(calcd for C_29_H_36_NO_2_, 430.2263). Myceliothermophin E (**2**), white amorphous powder, HRESIMS *m/z* 396.2897 [M + H]^+^ (calcd for C_26_H_38_NO_2_, 396.2903) (Yang Y.L. et al., 2007). Chaetolivacine B (**3**), yellow amorphous powder, ${\left[\alpha \right]}_{D}^{20}$ = + 28.5 (c 0.1, MeOH); HRESIMS *m/z* 462.3036 [M + H]^+^ (calcd for C_30_H_40_NO_3_, 462.3033). Chaetolivacine C (**4**), yellow amorphous powder, ${\left[\alpha \right]}_{D}^{20}$ = −18.8 (c 0.1, MeOH); HRESIMS *m/z* 462.2999 [M + H]^+^ (calcd for C_30_H_40_NO_3_, 462.3003).*

Compound **4** was also obtained as yellow amorphous powders. The molecular formula of **4** was designated as C_30_H_39_NO_3_ according to the ion peaks at *m/z* 462.2999 [M + H]^+^ in its HRESIMS (Supplementary Figure S18). The ^1^H and ^13^C NMR data of **4** (Supplementary Figures S19, S20) are highly similar to that of **3**, except for δ_H_ 7.40 (H-20) of **3** and δ_H_ 7.33 (H-20) of **4**. Compound **3**’s *Rt* = 15.0 min is different from **4**’s *Rt* = 17.6 min in HPLC (Supplementary Figure S25). The assignments of ^1^H and ^13^C signals of **4** are summarized in Table 3.

**FIGURE 5:**
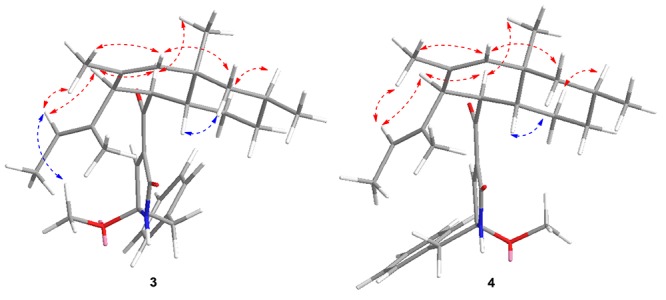
Key NOESY correlations of **3** and **4**.

Attentive analyses of HSQC, ^1^H–^1^H COSY, and HMBC spectra of **3** (Supplementary Figures S14–S16) and **4** (Supplementary Figures S21–S23) revealed that they have the same planar structures, which are proposed to be diastereomers. Spectral data indicated that **3** and **4** are a pair of isomers with different configurations. The observations also support the above deduction. However, based on the different chemical shifts of H-3, H-4, and the deoxy-tetramic acid moieties proposed, the configuration of C-21 in **3** and **4** is reversed (Yang Y.L. et al., 2007). In our situation, the NOESY correlation between H-15 and H-24 in **3** (Supplementary Figure S17) in the absence of the correlation in **4** (Supplementary Figure S24) suggests the relative configuration of C-21. Thus, the relative configurations of **3** and **4** were determined as 3*S*^∗^4*R*^∗^5*R*^∗^8*R*^∗^10*S*^∗^21*S*^∗^ and 3*S*^∗^4*R*^∗^5*R*^∗^8*R*^∗^10*S*^∗^21*R*^∗^, respectively (Figure 5).

The absolute configuration of **3** and **4** was further elucidated by comparing the experimental ECD spectra with those calculated at the b3lyp/6-311 + g (d, p) and b3lyp/6-31 + g (d, p) levels. The results suggest that they were epimers at C-21 while both of them perform the absolute configuration in deoxy-tetramic acid moieties at 3*S*, 4*R*, 5*R*, 8*R*, 10*S* (Yang Y.L. et al., 2007).

### Antimicrobial Activity of Compounds

The isolated compounds **1**–**4** were evaluated for their antimicrobial properties, specifically their abilities against drug-resistant bacteria. Their antimicrobial activity against SA, MRSA, G5, SC5314, 17#, and PA14 were evaluated. As shown in the results, compounds **1**–**4** exhibited weak inhibitive activity against SC5314, 17#, and G5 as well as PA14; however, compounds **2** and **3** showed inhibitive activity against SA and MRSA (Table 4). Compound **3** showed moderate anti-SA bioactivity with an MIC of 10.8 μM. Compounds **2** and **3** showed potent inhibitory effects against MRSA with MIC values of 15.8 and 27.1 μM. For the isomers 21*R*-**3** and 21*S*-**4**, the MIC value of inhibition of SA and MRSA increased from 10.8 and 27.1 μM, respectively, to over 100 μM, implying that configurations of C-21 played a critical role in the biological activity toward SA and MRSA.

**TABLE 4:** Antimicrobial activity of compounds **1**–**4**.

**Compound**	**SA (MIC/μM)**	**MRSA (MIC/μM)**	**G5 (MIC/μM)**	**SC5314 (MIC/μM)**	**17# (MIC/μM)**	**PA14 (MIC/μM)**
1	>100	>100	>100	>100	>100	>100
2	>100	15.8	>100	>100	>100	>100
3	10.8	27.1	>100	>100	>100	>100
4	>100	>100	>100	>100	>100	>100

*Vancomycin (McCormick et al., 1995) was positive control of SA and MRSA, MIC = 0.84 μM. Ciprofloxacin as positive control of PA, MIC = 0.19 μM; amphotericin B, positive control for SC5314, 17#, and G5, MIC = 1.69 μM.*

### Postulated Biogenetic Pathways

**FIGURE 6:**
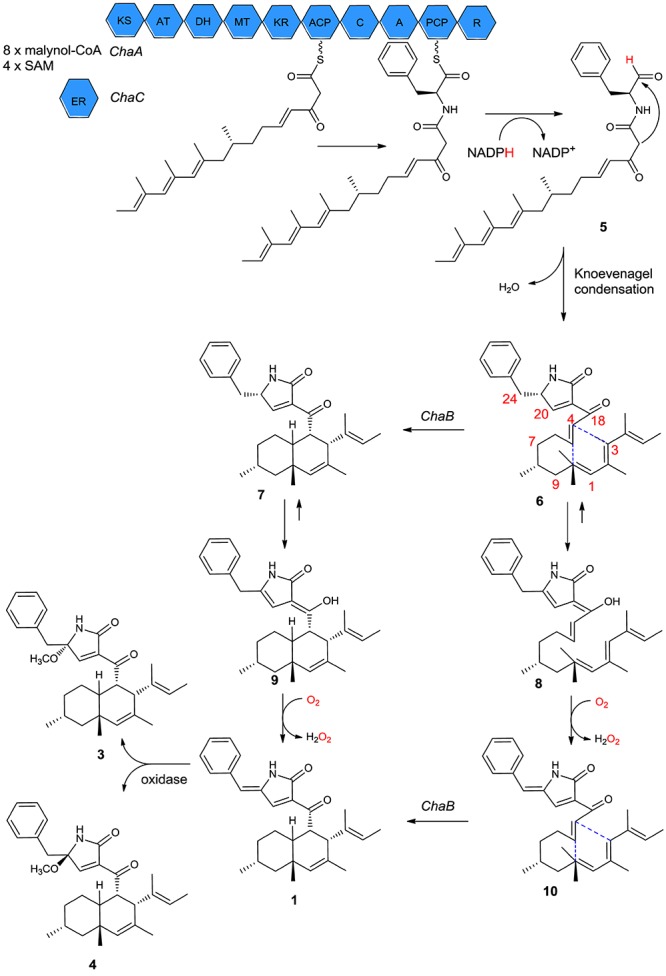
Proposed biosynthetic pathway for compound **1** based on isolated natural products and biochemical characterization of *ChaB*.

It has been reported that some secondary metabolites such as oteromycin (Singh et al., 1995; Uchiro et al., 2013), ZG-1494a (West et al., 1996), UCS1025 A and B (Agatsuma et al., 2002; Lambert and Danishefsky, 2005), trichosetin (Marfori et al., 2002), and talaroconvolutins A–D (Suzuki et al., 2000) have a subtle biogenetic pathway in the deoxy-tetramic acid ring system. Although the pathways of the newly isolated compounds **1**, **3**, and **4** remain unclear, a reasonable biological pathway could be assumed. The three genes *ChaA*, *ChaB*, and *ChaC* may play an important role in the synthesis of compounds **1**–**4** (Li et al., 2016). The proteins encoded by *ChaA* and *ChaC* functioned with aminoacyl polyketide aldehyde **5** from eight malonyl-CoAs and four *S*-adenosylmethionines (SAM). Following *ChaA*-catalyzed constructions and the release of aminoacyl polyketide aldehyde **5**, then Knoevenagel condensation yielded the intermediate ketone **6**; as a substrate, **6** could lead to the formation of the endo product **7** by *ChaB*, and **7** will be used as a substrate to get **9** through enolization. Finally, **9** would be oxidized to form **1**. Another possible biosynthesis pathway of **1** is the creation of **6** by tautomerization, oxidation, and cyclization (Figure 6). Compounds **3** and **4** could be formed by the hydroxyl attached to C-21 from different directions of the double bond planar and that are then methylated. The pathway involves leucine in **2** or phenylalanine in **1**, **3**, and **4** that participates in a polyketide reaction.

Drug-resistant bacteria are microorganisms that produce tolerance to corresponding antibiotics after a long period of antibiotic selection. MRSA has been recognized as one of the major pathogens that cause nosocomial infections (Imamura et al., 2001). *Chaetomium*, which belongs to the family of Chaetomiaceae, contains >100 species derived from marine and terrestrial environments. In previous investigations, *Chaetomium* producing >200 bioactive metabolites has been a valuable source for mining anti-MRSA compounds (Zhang et al., 2012). Our study clearly shows that *C. olivaceum* hosts rich genes, including I PKS, NRPS, PKS-NRPS, terpene, and other kinds. However, *trans*-decalin ring systems were found in *Chaetomium* for the first time. In this study, compounds **1**–**4** containing naphthalene type tetramic acid groups produced by *C. olivaceum* SD-80A were obtained. The terminal of the tetramic acid group in compounds **1**, **3**, and **4** were identical benzene rings. Compounds **3** and **4** were identified as isomers with different configurations of C-21. These compounds were tested against drug-resistant bacteria and revealed moderate activity, and the configuration of C-21 is important for anti-MRSA activity in these kind of compounds. As reported before, the configuration of C-21 in the two sets of diastereomers of myceliothermophins A–E also play a critical role in the biological toxicities toward cancer cells (Yang Y.L. et al., 2007). The results revealed the significant potential of the genus *Chaetomium* in producing new compounds with PKS-NRPS, which will speed the mining for new sources of antimicrobial agents.

Genome mining has recently been used for the discovery of new secondary metabolites. Cycloaddition reactions such as IMDA reactions are found to occur widely among fungal natural product pathways of PKS and NRPS (Minami and Oikawa, 2016), like lovastatin (Auclair et al., 2000) and cytochalasin (Scherlach et al., 2010). Therefore, using DAases (MycB) as biosynthesis marker to focus *C. olivaceum* SD-80A efficiently from our fungi library, then decalin-containing natural products **1**–**4** were obtained, and these compounds involved three proteins encoded by key genes *ChaA* responding to the synthesis of the acetate-derived portion, including the decalin. Concurrently, the NRPS adds the amino acid *ChaB*, which responds for a Diels–Alder reaction, and *ChaC*, an auxiliary enoyl reductase (ER) is created. The predicted biosynthesis pathway provided a theoretical basis for the future biosynthesis of these compounds using a heterologous expression. For compounds **1**–**4**, leucine in **2** or phenylalanine in **1**, **3**, and **4** involved in the biosynthesis pathway **2** with isopropyl showed stronger bioactivity than **1** with phenyl against MRSA with an MIC of 15.8 μM and over 100, respectively. Therefore, the different groups from amino acid as a precursor may play an important role in the antimicrobial activity of this kind of compound. The experiments for more novel bioactive compounds of this class using different amino acid as a precursor are ongoing.

## Conclusion

In this study, using genome mining and *ChaB* as a probe, compounds **1**–**4** were isolated as polyketones containing naphthalene-type tetramic acid groups produced by *C. olivaceum* SD-80A. This is the first report of this kind in regard to compounds generated from *Chaetomium*. The terminal of the tetramic acid group in compounds **1**, **3**, and **4** were identical benzene rings, and compounds **3** and **4** were identified as isomers with different configurations of C-21. These compounds were tested against drug-resistant bacteria and revealed moderate activity, and configurations of C-21 are important for anti-MRSA activity in this kind of compound. The biosynthetic pathway was proposed, which provides a theoretical basis for the future biosynthesis of these compounds in *C. olivaceum* SD-80A. This also presents an interesting potential application in food, feed, as well as pharmaceutical industries. The experiments for more novel bioactive compounds of this class using different amino acid as precursor are ongoing.

## Data Availability Statement

All datasets generated for this study are included in the article/Supplementary  Material.

## Author Contributions

XX, ZXL, and LXZ conceived, designed, and coordinated the study, edited the manuscript, and critically revised the manuscript. XW contributed to the extraction and purification of the compounds, data acquisition, analysis, and interpretation, and manuscript drafting. LYZ did the bioinformatics, genome analysis, and helped in the manuscript writing. ZML did the chemical calculation. JQ and XL helped with the experimental assays and the manuscript revision. PZ, CLL, and XY helped with the antimicrobial experiments and verifying the identity of all the strains. CL and YH contributed to the manuscript preparation.

## Conflict of Interest

The authors declare that the research was conducted in the absence of any commercial or financial relationships that could be construed as a potential conflict of interest.

## Footnotes

1 https://blast.ncbi.nlm.nih.gov/Blast.cgi
