# Supplementary material for: New Tetramic Acids Comprising of Decalin and Pyridones From Chaetomium olivaceum SD-80A With Antimicrobial Activity
Source: Front Microbiol. 2020 Jan 15;10:2958. doi: 10.3389/fmicb.2019.02958 (PMC6974552; doi:10.3389/fmicb.2019.02958)
Supplement: Supplementary file 1 [file Data_Sheet_1.docx]

**Supporting Information**

**New Tetramic Acids Comprising of Decalin and Pyridones from *Chaetomium Olivaceum* SD-80A with Antimicrobial Activity**

Xinzhu Wang^1^, Liya Zhao^2^, Chao Liu^3^, Jun Qi^2^, Peipei Zhao^2^, Zhaoming Liu^5^, Chunlei Li^2^, Yingying Hu^1^, Xin Yin^2^, Xin Liu^2^, Zhixin Liao^1*^, Lixin Zhang^2,4*^, Xuekui Xia^2*^

^1^Department of Pharmaceutical Engineering, School of Chemistry and Chemical Engineering, Southeast University, Nanjing 211189, China

^2^Key Laboratory for Biosensor of Shandong Province, Biology Institute, Qilu University of Technology (Shandong Academy of Sciences), Jinan 250014, China

^3^Institute of Agro-Food Science and Technology, Shandong Academy of Agricultural Sciences, 202 Gongye North Road, Jinan 250100, China.

^4^State Key Laboratory of Bioreactor Engineering, East China University of Science and Technology, Shanghai 200237, China

^5^Guangdong Institute of Microbiology Guangdong Academy of Sciences, 100 Central Xianlie Road, Yuexiu District, Guangzhou 510070, China

* Corresponding author *Email:*

Zhixin Liao

zxliao@seu.edu.cn (Z.X. Liao)

Xuekui Xia

xiaxk@sdas.org (X.K. Xia)

Lixin Zhang

lxzhang@ecust.edu.cn (L.X. Zhang).

**Contents**

[**Figure S1**. The HRESIMS of compound **1** 3](#_Toc10927916)

[**Figure S2**. The ^1^H NMR (400 MHz, CDCl_3_) spectrum of compound **1** 4](#_Toc10927917)

[**Figure S3**. The ^13^C NMR (100 MHz, CDCl_3_) spectrum of compound **1** 5](#_Toc10927918)

[**Figure S4**. The COSY (400 MHz, CDCl_3_) spectrum of compound **1** 6](#_Toc10927919)

[**Figure S5**. The HSQC (400 MHz, CDCl_3_) spectrum of compound **1** 7](#_Toc10927920)

[**Figure S6**. The HMBC (400 MHz, CDCl_3_) spectrum of compound **1** 8](#_Toc10927921)

[**Figure S7**. The NOESY (400 MHz, CDCl_3_) spectrum of compound **1** 9](#_Toc10927922)

[**Figure S8**. The ESIMS of compound **2** 10](#_Toc10927923)

[**Figure S9**. The ^1^H NMR (400 MHz, CDCl_3_) spectrum of compound **2** 11](#_Toc10927924)

[**Figure S10**. The ^13^C NMR (100 MHz, CDCl_3_) spectrum of compound **2** 13](#_Toc10927925)

[**Figure S11**. The HRESIMS of compound **3** 13](#_Toc10927926)

[**Figure S12**. The ^1^H NMR (400 MHz, CDCl_3_) spectrum of compound **3** 14](#_Toc10927927)

[**Figure S13**. The ^13^C NMR (100 MHz, CDCl_3_) spectrum of compound **3** 15](#_Toc10927928)

[**Figure S14**. The COSY (400 MHz, CDCl_3_) spectrum of compound **3** 16](#_Toc10927929)

[**Figure S15**. The HSQC (400 MHz, CDCl_3_) spectrum of compound **3** 17](#_Toc10927930)

[**Figure S16**. The HMBC (400 MHz, CDCl_3_) spectrum of compound **3** 18](#_Toc10927931)

[**Figure S17**. The NOESY (400 MHz, CDCl_3_) spectrum of compound **3** 19](#_Toc10927932)

[**Figure S18**. The HRESIMS of compound **4** 20](#_Toc10927933)

[**Figure S19**. The ^1^H NMR (400 MHz, CDCl_3_) spectrum of compound **4** 21](#_Toc10927934)

[**Figure S20**. The ^13^C NMR (100 MHz, CDCl_3_) spectrum of compound **4** 22](#_Toc10927935)

[**Figure S21**. The COSY (400 MHz, CDCl_3_) spectrum of compound **4** 23](#_Toc10927936)

[**Figure S22**. The HSQC (400 MHz, CDCl_3_) spectrum of compound **4** 24](#_Toc10927937)

[**Figure S23**. The HMBC (400 MHz, CDCl_3_) spectrum of compound **4** 25](#_Toc10927938)

[**Figure S24**. The NOESY (400 MHz, CDCl_3_) spectrum of compound **4** 26](#_Toc10927939)

[**Figure S25**. Semi-preparative HPLC method of isolating compounds **3** (15.0 min) and **4** (17.6 min) 27](#_Toc10927940)

**Figure S1**. The HRESIMS of compound **1**


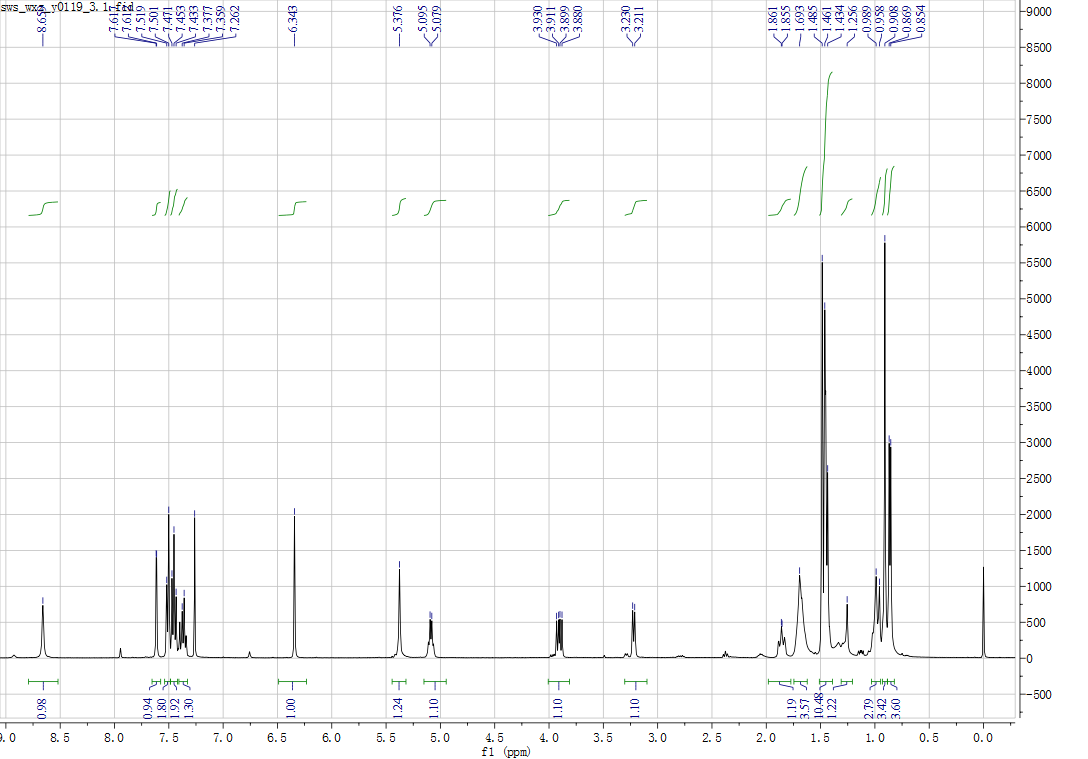


**Figure S2**. The ^1^H NMR (400 MHz, CDCl_3_) spectrum of compound **1**


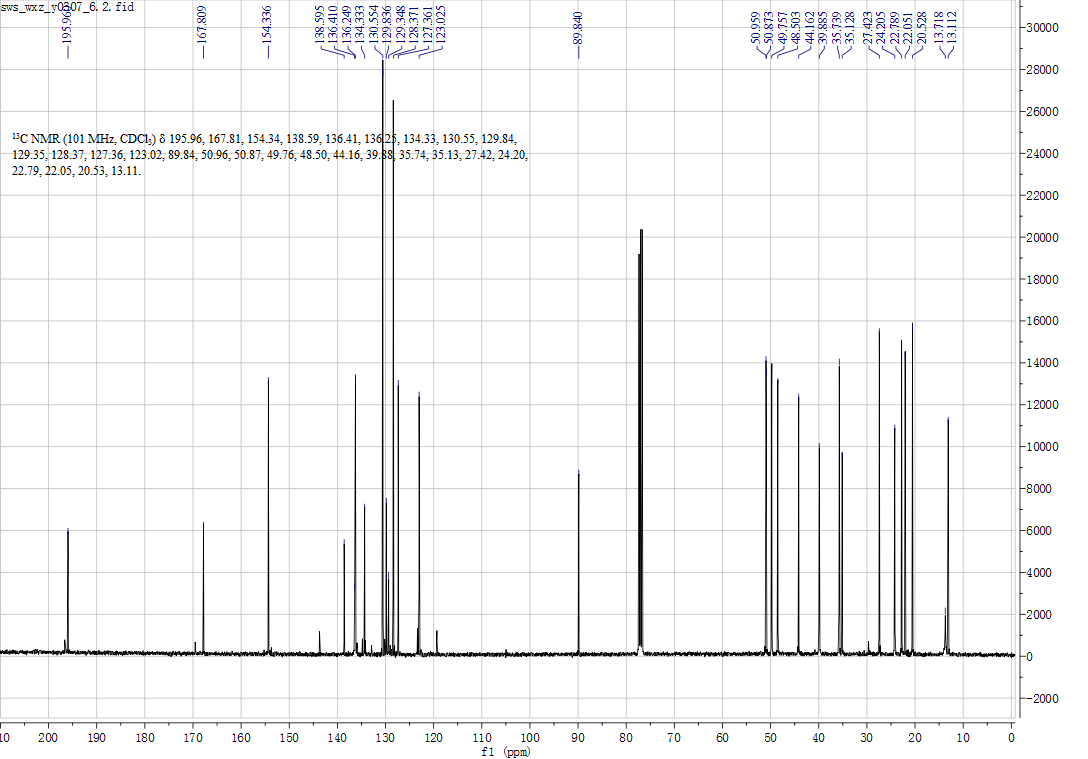


**Figure S3**. The ^13^C NMR (125 MHz, CDCl_3_) spectrum of compound **1**


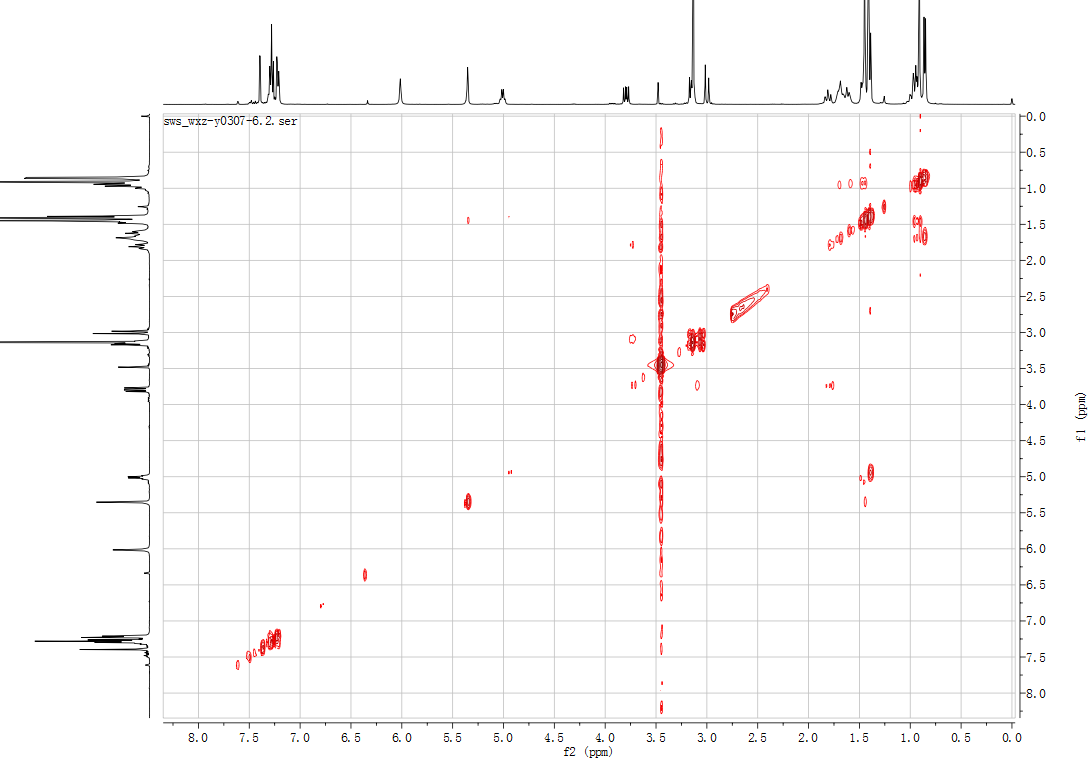


**Figure S4**. The COSY (400 MHz, CDCl_3_) spectrum of compound **1**

**
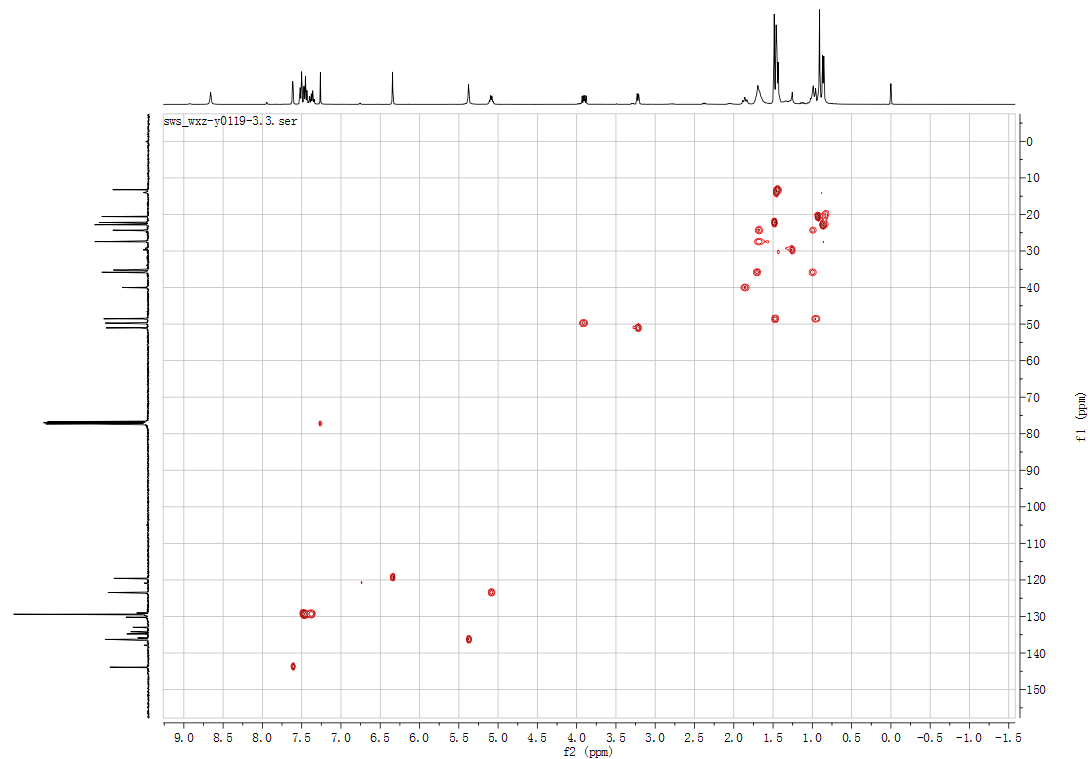
**

**Figure S5**. The HSQC (400 MHz, CDCl_3_) spectrum of compound **1**

**
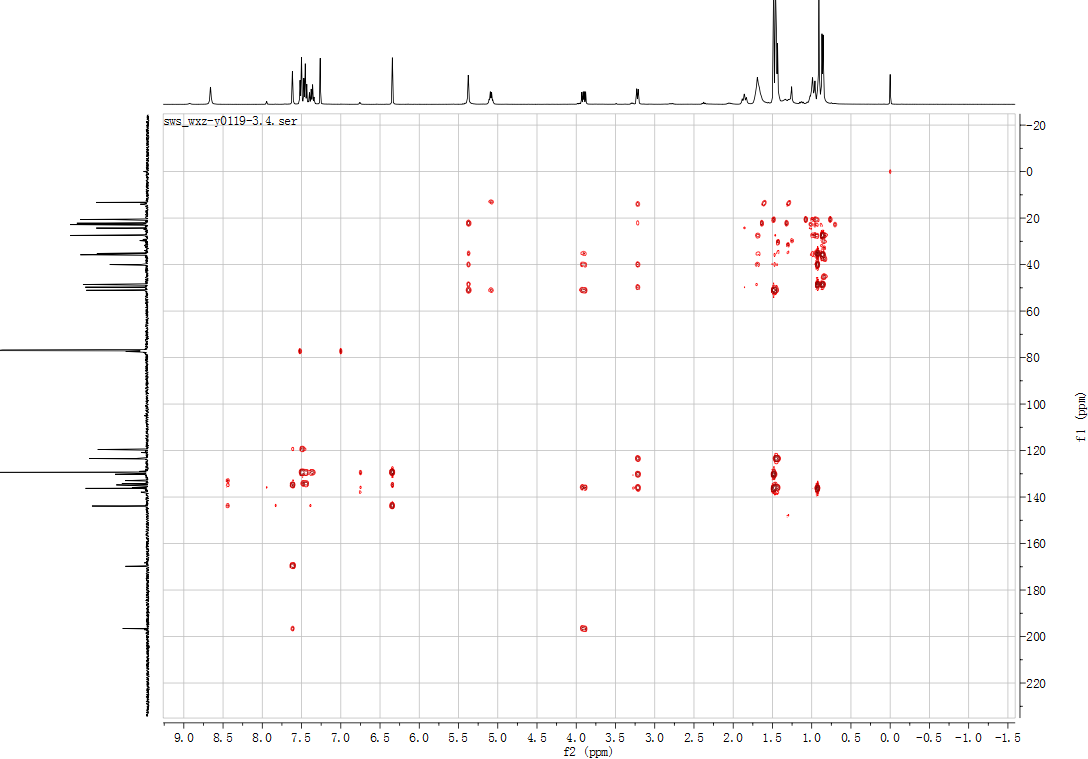
**

**Figure S6**. The HMBC (400 MHz, CDCl_3_) spectrum of compound **1**

**
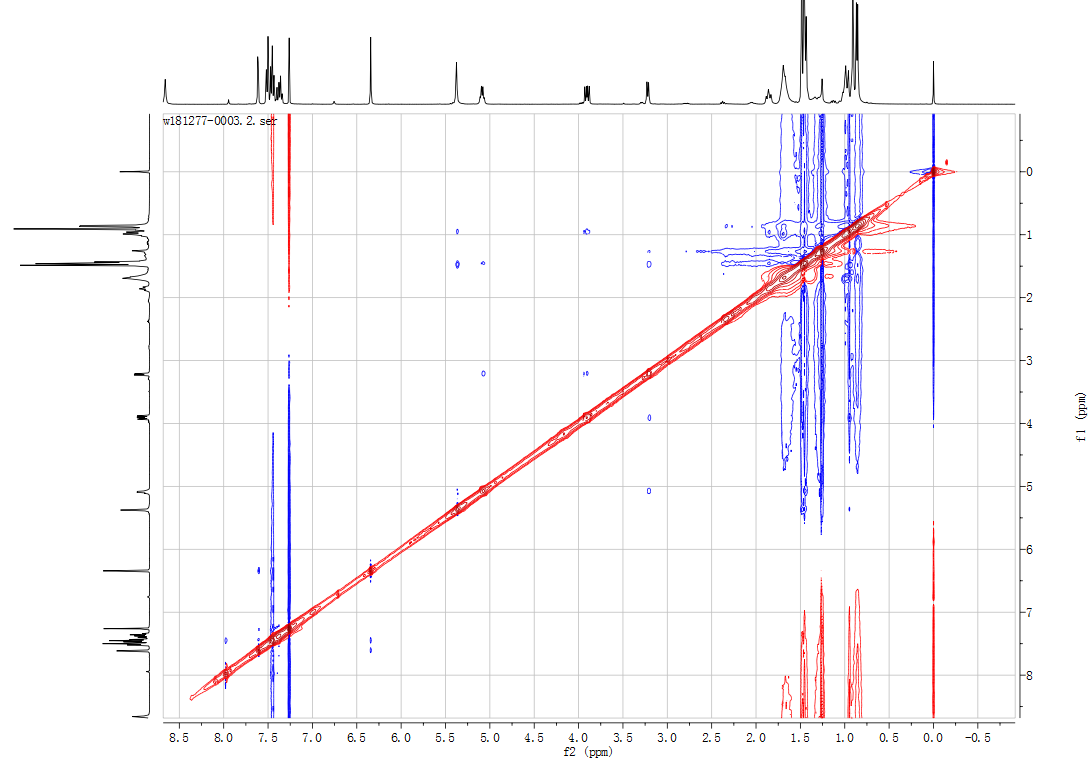
**

**Figure S7**. The NOESY (400 MHz, CDCl_3_) spectrum of compound **1**

**Figure S8**. The ESIMS of compound **2**

**
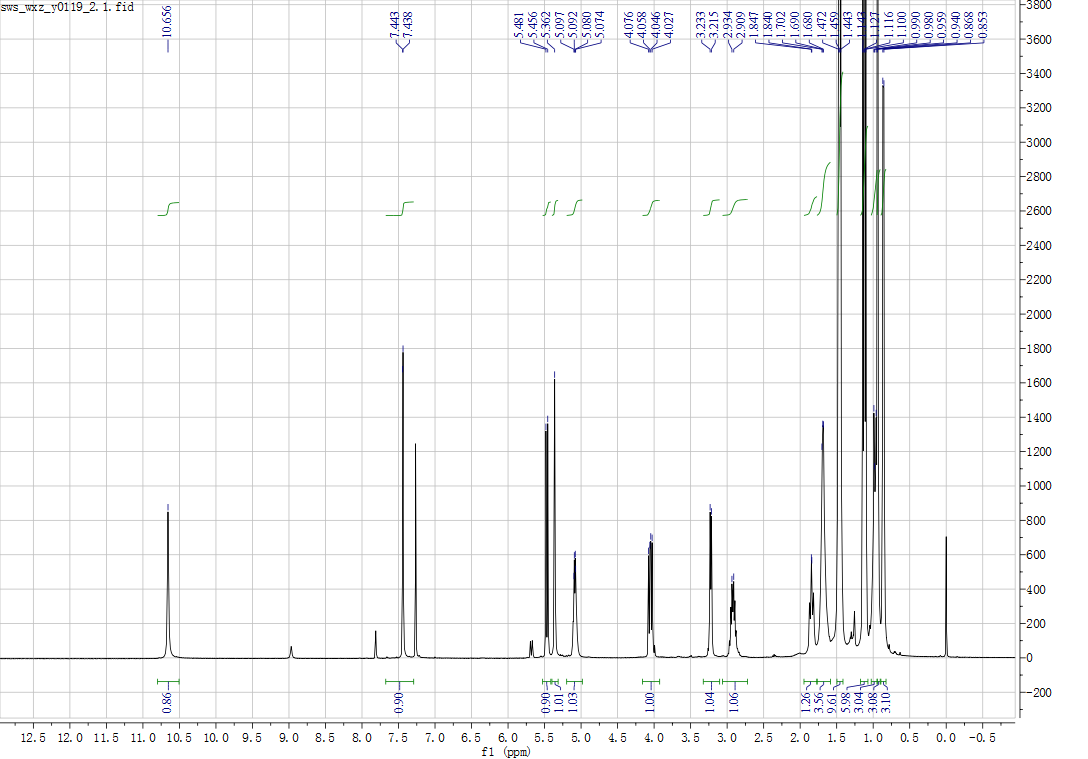
**

**Figure S9**. The ^1^H NMR (400 MHz, CDCl_3_) spectrum of compound **2**

**
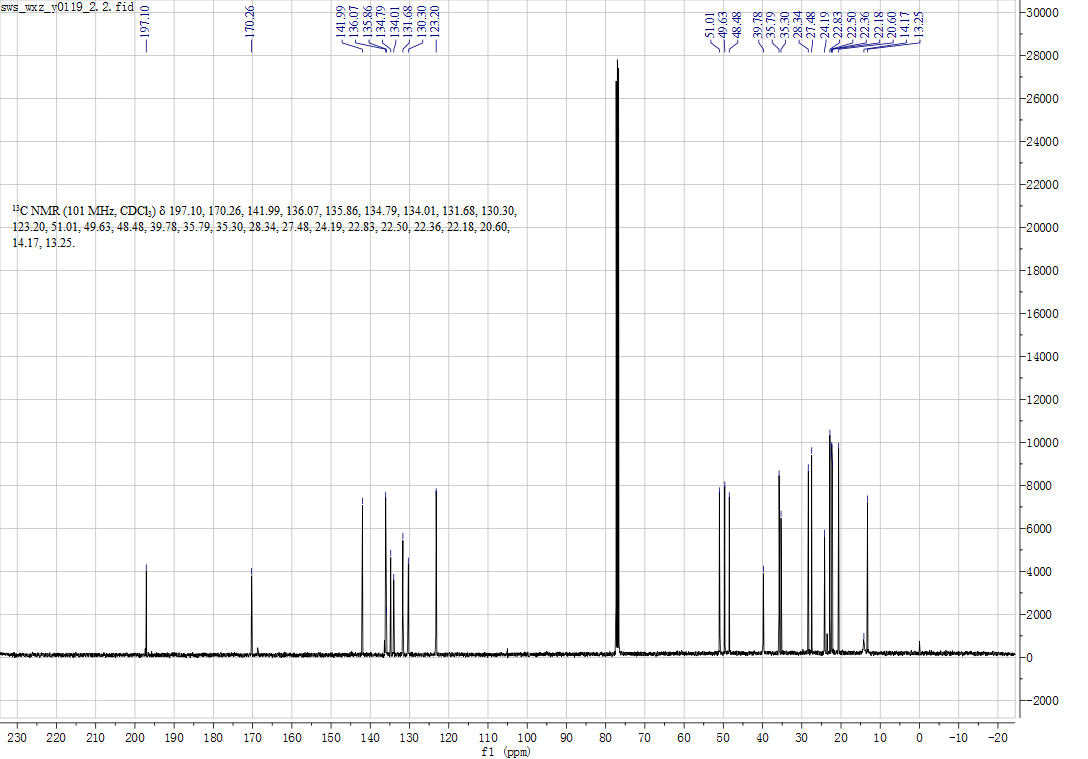
**

**Figure S10**. The ^13^C NMR (125 MHz, CDCl_3_) spectrum of compound **2**

**Figure S11**. The HRESIMS of compound **3**


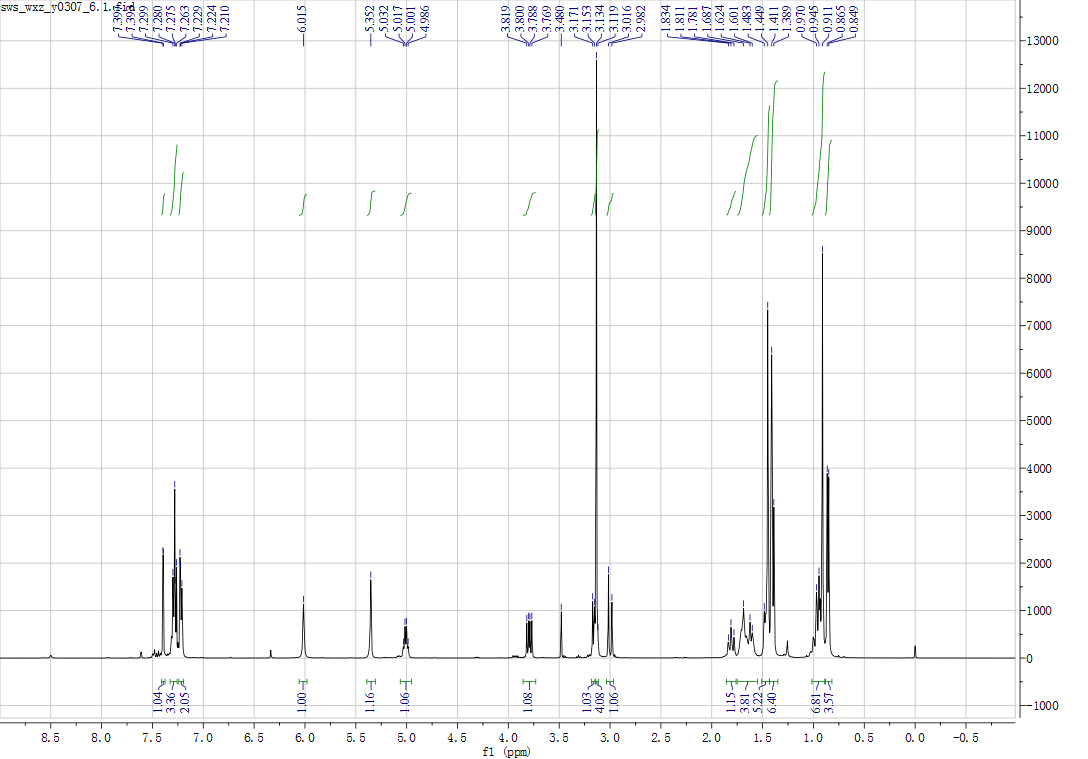


**Figure S12**. The ^1^H NMR (400 MHz, CDCl_3_) spectrum of compound **3**

**
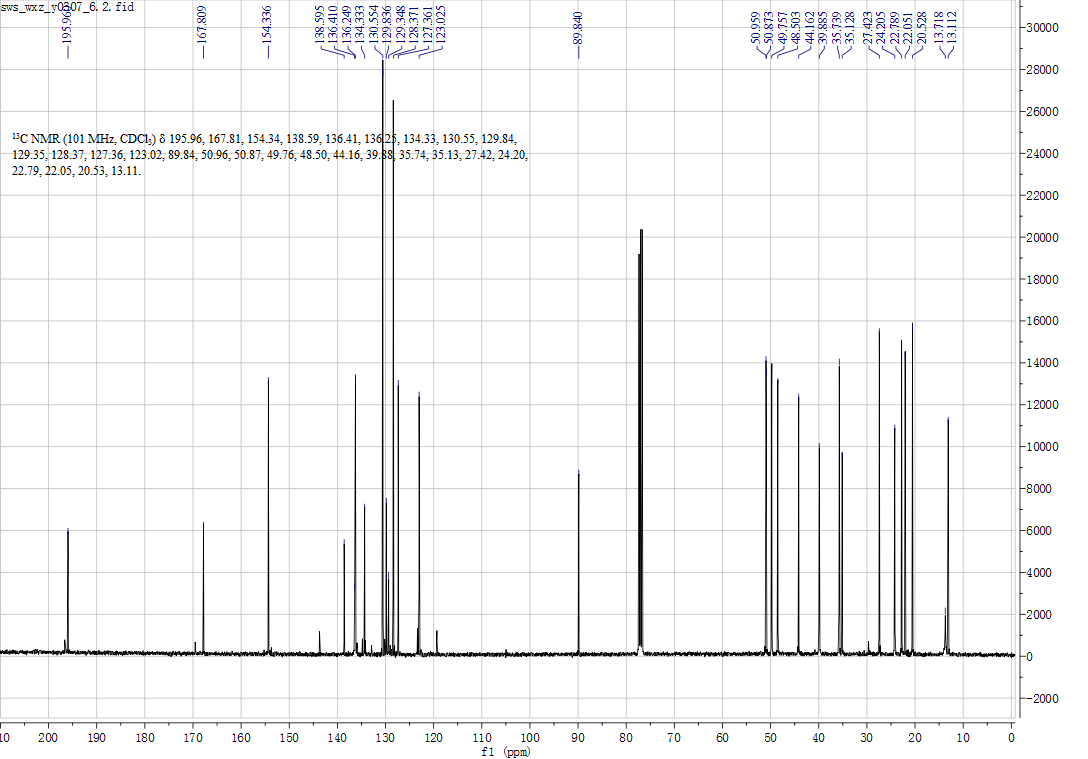
**

**Figure S13**. The ^13^C NMR (125 MHz, CDCl_3_) spectrum of compound **3**

**
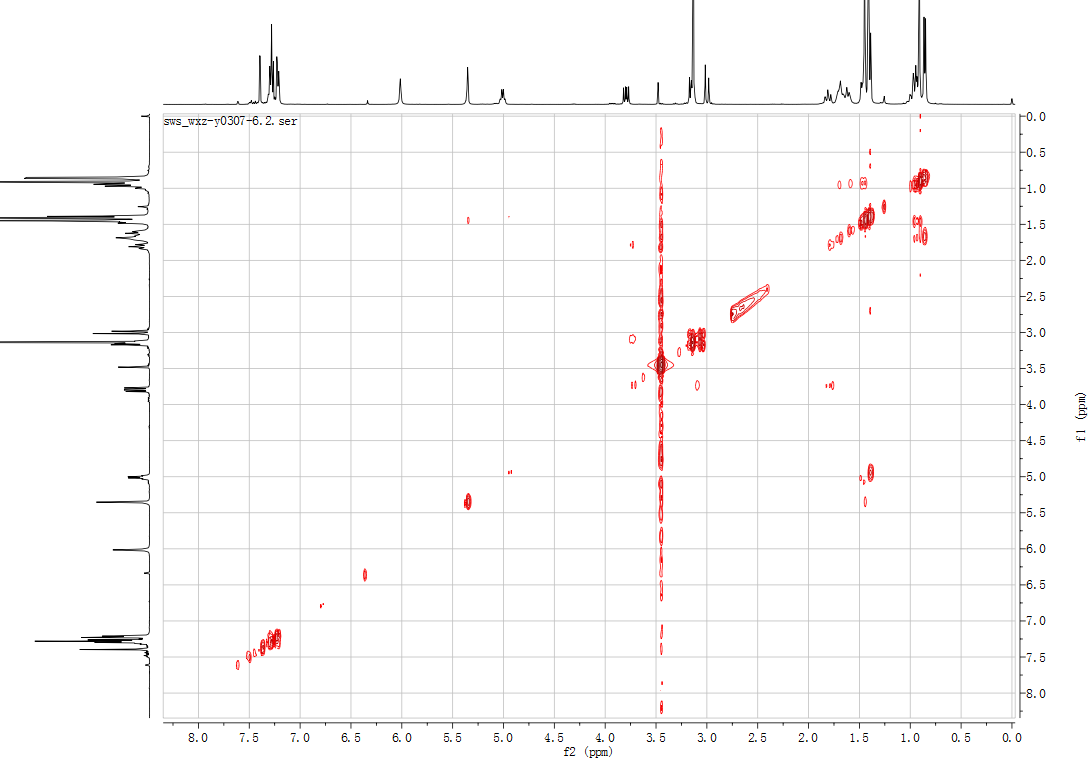
**

**Figure S14**. The COSY (400 MHz, CDCl_3_) spectrum of compound **3**


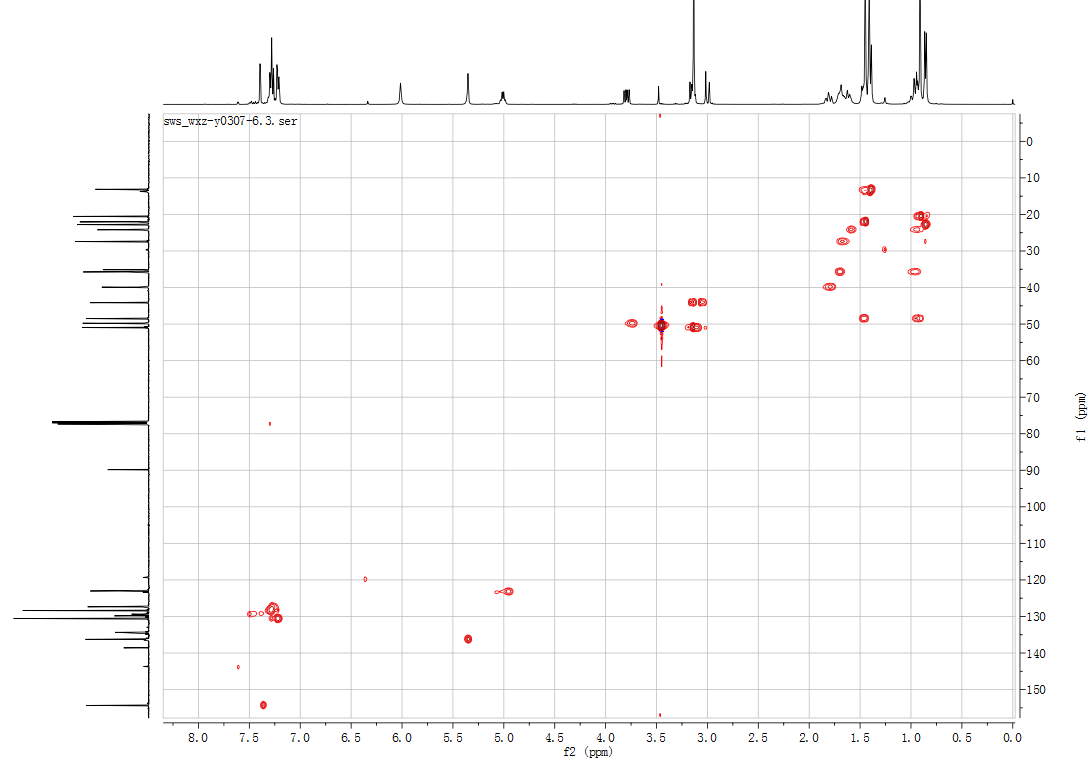


**Figure S15**. The HSQC (400 MHz, CDCl_3_) spectrum of compound **3**


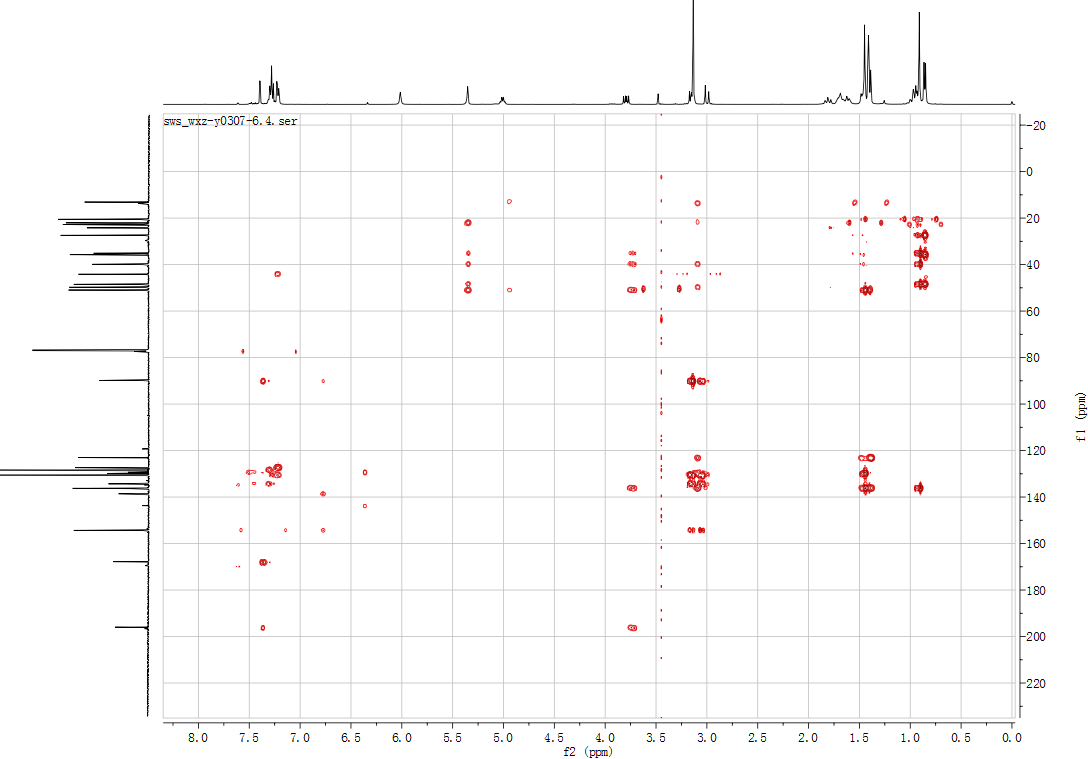


**Figure S16**. The HMBC (400 MHz, CDCl_3_) spectrum of compound **3**


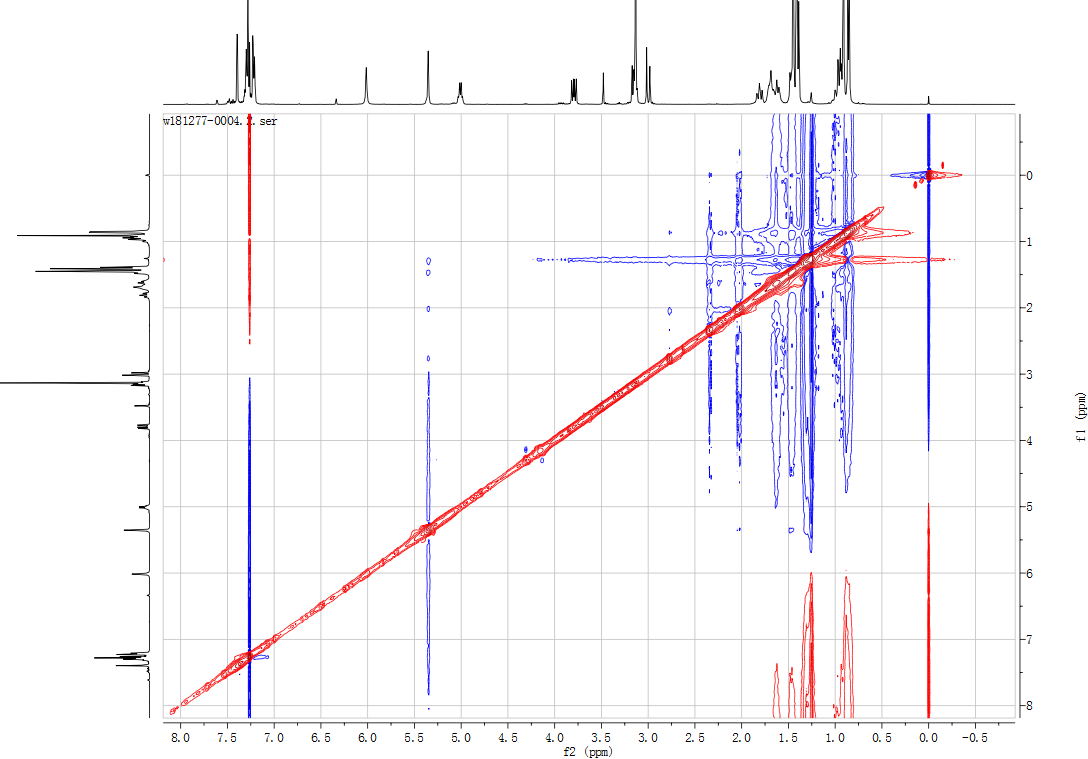


**Figure S17**. The NOESY (400 MHz, CDCl_3_) spectrum of compound **3**

**Figure S18**. The HRESIMS of compound **4**


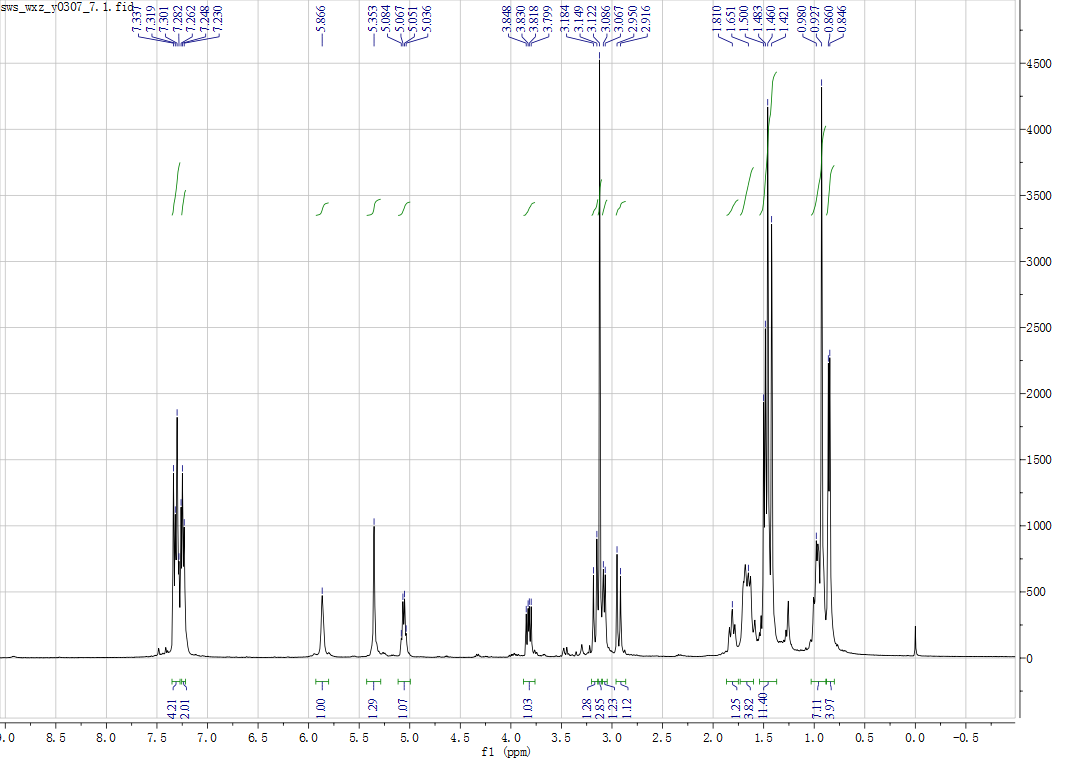


**Figure S19**. The ^1^H NMR (400 MHz, CDCl_3_) spectrum of compound **4**


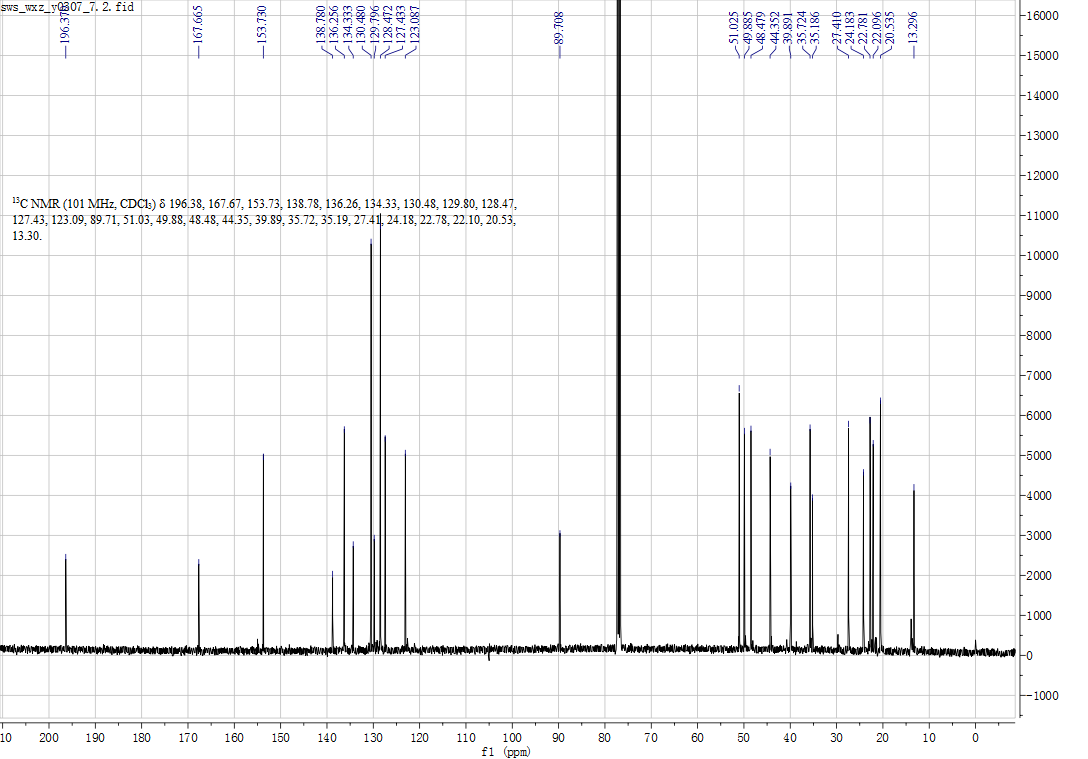


**Figure S20**. The ^13^C NMR (125 MHz, CDCl_3_) spectrum of compound **4**


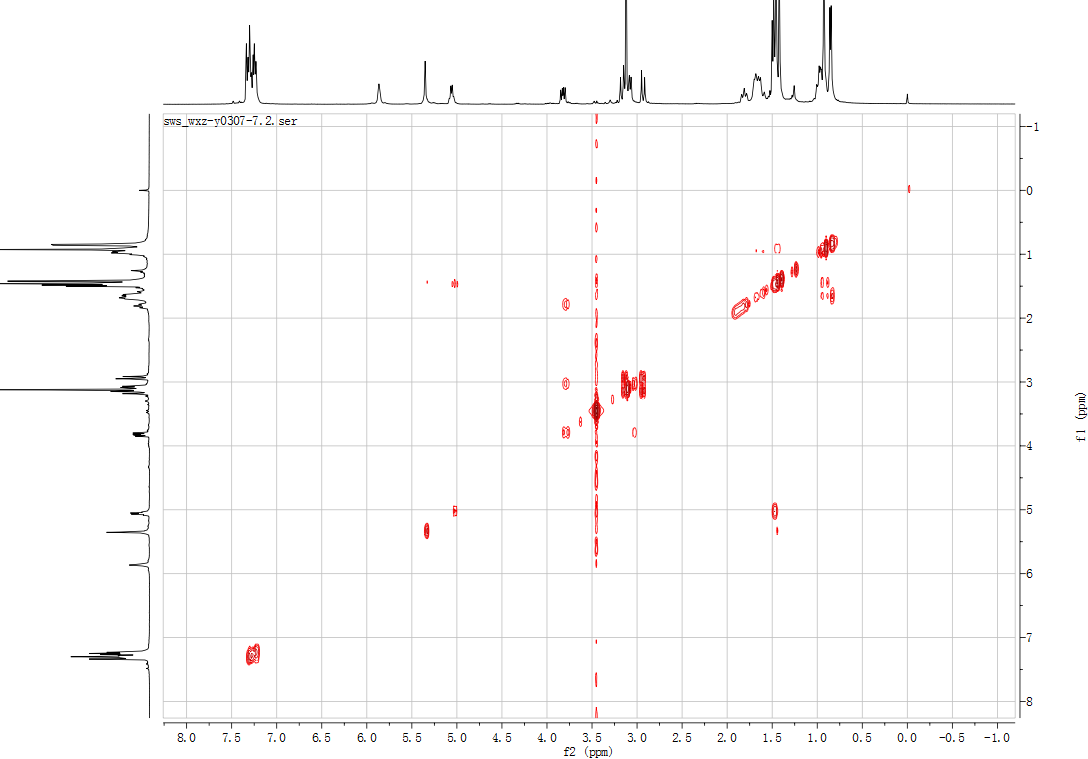


**Figure S21**. The COSY (400 MHz, CDCl_3_) spectrum of compound **4**

**
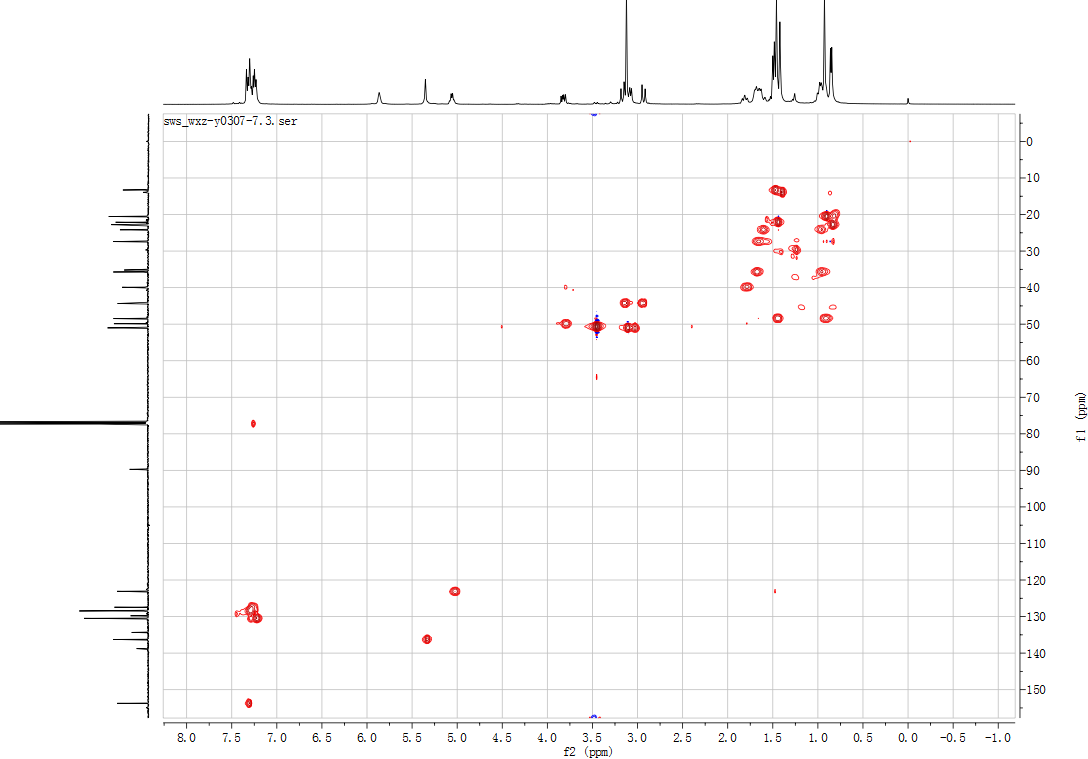
**

**Figure S22**. The HSQC (400 MHz, CDCl_3_) spectrum of compound **4**


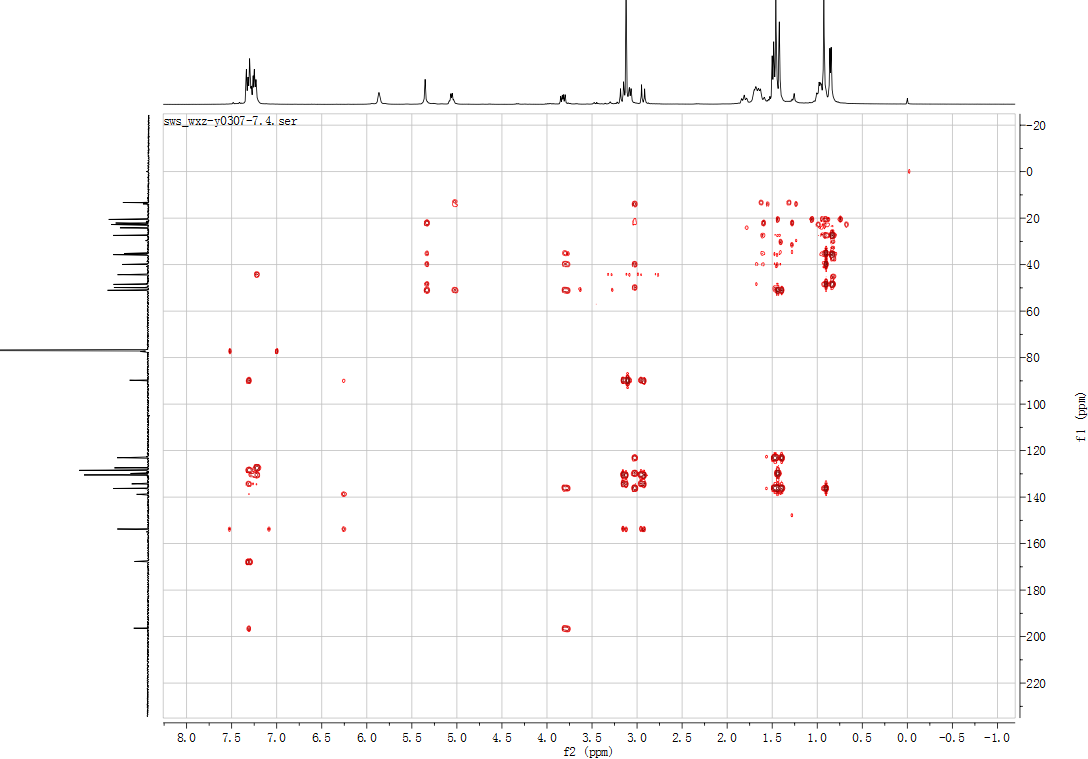


**Figure S23**. The HMBC (400 MHz, CDCl_3_) spectrum of compound **4**


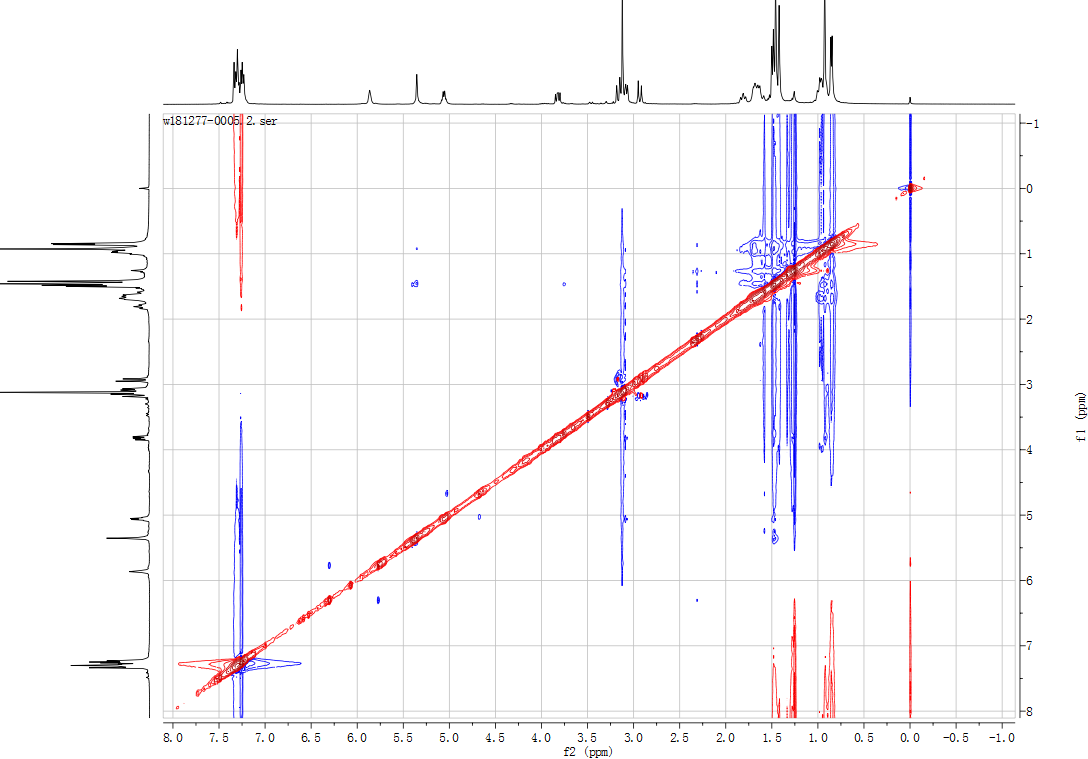


**Figure S24**. The NOESY (400 MHz, CDCl_3_) spectrum of compound **4**


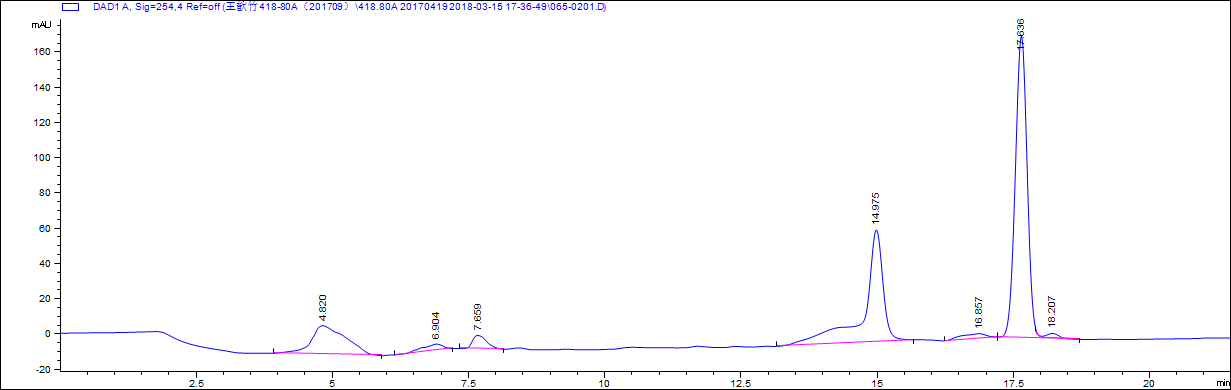


**Figure S25**. Semi-preparative HPLC method of isolating compounds **3** (15.0 min) and **4** (17.6 min)
